# Decreased incidence of glaucoma in children with asthma using inhaled corticosteroid: a cohort study

**DOI:** 10.18632/oncotarget.22252

**Published:** 2017-11-01

**Authors:** Ling-Sai Chang, Hui-Ching Lee, Yuh-Chyn Tsai, Lien-Shi Shen, Ching-Ling Li, Shih-Feng Liu, Ho-Chang Kuo

**Affiliations:** ^1^ Kawasaki Disease Center, Kaohsiung Chang Gung Memorial Hospital, Taiwan, China; ^2^ Graduate Institute of Clinical Medical Sciences, College of Medicine, Chang Gung University, Taiwan, China; ^3^ Department of Pediatrics, Kaohsiung Chang Gung Memorial Hospital and Chang Gung University College of Medicine, Taiwan, China; ^4^ Department of Respiratory Therapy, Kaohsiung Chang Gung Memorial Hospital, Taiwan, China; ^5^ Division of Pulmonary and Critical Care Medicine, Department of Internal Medicine, Kaohsiung Chang Gung Memorial Hospital and Chang Gung University College of Medicine, Taiwan, China

**Keywords:** asthma, child, glaucoma, inhaled corticosteroid, taiwan national health

## Abstract

Among the anti-inflammatory medications used for treating asthma, corticosteroids are the most effective. The effects of orally administered corticosteroids on intraocular pressure and lens opacity have been well defined, but the influence of inhaled corticosteroids (ICS) on children has yet to be clearly explained. Therefore, we used a nationwide cohort database to investigate glaucoma in childhood asthma patients using ICS. We analyzed a dataset of 1,000,000 randomly sampled individuals from Taiwan's 2000 National Health Insurance Research Database. The study cohort included 5,380 patients who were first diagnosed with asthma (ICD9: 493.X) diagnosis when they were six years old or younger. All subjects were followed through December 2011. We applied Cox's proportional hazard model to determine whether ICS use has a correlation with glaucoma. Of the 5,380 patients enrolled in this study, we identified 1,232 patients who had used ICS and 4,148 patients who had no history of ICS administration throughout the follow-up period. The prevalence of glaucoma was significantly lower in patients using ICS, with a 0.52-fold decreased risk of developing glaucoma in comparison to the control group [adjusted hazard ratio (HR) 0.52, 95% confidence interval (CI) 0.28∼0.96]. Among the evaluated comorbidities, cataract was positively associated with glaucoma in asthma children (adjusted HR 8.22; 95% CI = 2.59∼26.12). This study provides not only the first but also strong evidence that the glaucoma incidence in the ICS group is lower than that in the non-ICS group in children with asthma. Further consultation with an ophthalmologist regarding the high-risk group of asthma children with cataracts is necessary.

## INTRODUCTION

Asthma is one of the most widespread chronic childhood diseases, often starting in children under the age of six years [1]. This disease is commonly treated with corticosteroids. Short-burst oral corticosteroids are frequently considered for treating young children admitted to the hospital with symptoms indicating atopic asthma [2]. Meanwhile, inhaled corticosteroids (ICS) can effectively treat multiple-trigger wheeze in preschool-age children [3].

Side effects caused by ICS usage are particularly important when taken by children because of the disruption to the hypothalamic-pituitary-adrenal axis function, bone turnover, osteoporosis, and growth suppression [4]. Furthermore, such adverse effects as candidiasis, cataracts, glaucoma, and osteoporosis, which may be caused by inhaled corticosteroids in the elderly, are quite concerning, as well [5]. As for systemic corticosteroids, the increased risk of developing glaucoma has been well documented [6]. Decreased trabecular meshwork outflow is regarded as the primary cause of steroid-induced iatrogenic glaucoma [6]. ICS were created to target their delivery into the lungs, which reduces the systemic side effects commonly caused by oral corticosteroids. In fact, the safety profile of all ICS preparations is considerably better than that of oral corticosteroids. Using ICS therapy to treat asthma does not influence the risk of developing glaucoma in adults [5]. However, in an observational case series that included five children with leukemia under the age of six years, elevated intraocular pressure was seen in all those who received systemic steroids [7]. Kwok et al. found that the steroid intraocular pressure-increasing effect was greater in children under the age of ten years with topical dexamethasone [8]. However, little has been discovered with regard to the likelihood of developing glaucoma in preschool-age children, especially concerning inhaled corticosteroids. The importance of the topic and scarceness of relevant data led us to explore the correlation between glaucoma and ICS in young children.

In Taiwan, the National Health Insurance (NHI) program has been covering nearly all of Taiwan's inhabitants since 1996, and all claims data are gathered by the National Health Insurance Research Database (NHIRD) [9]. Currently, the complete database may be one of the largest such health insurance databases in the world. As a result, researchers can easily conduct a nationwide medical use study using this highly computerized database. In this study, we used data from Taiwan's NHIRD to evaluate the relationship between glaucoma and ICS use.

## RESULTS

### Patient characteristics of ICS users and non-ICS users

This study cohort included the data of 91,363 children under the age of six years old from 1997 to 2001. Of those, 5,386 children were admitted to the hospital or visited outpatient clinics more than three times for asthma during the study period. We excluded children that had been previously diagnosed with glaucoma (n=6), resulting in 5,380 patients, including 1,232 ICS users and 4,148 non-ICS users, being used in our final analysis. Differences in income and comorbidities like prematurity, myopia, cataract, and diabetes were eliminated between the ICS users and non-users (all p-values > 0.05). Table 1 shows the demographic characteristics of ICS users versus non-ICS users. Our sample had significantly fewer ICS-users than non-ICS users (1,232 vs. 4,148), and male patients were found to have a higher rate of ICS usage (798/3309 vs. 434/2071, p=0.0073) than female patients. The median age of patients was 3.12 years old, and ICS was prescribed more frequently for children above the age of 3.12 years old (53.9% vs. 46.1%, p=0.0025).

**Table 1:** Characteristics of ICS and non-ICS users in preschool-age children with asthma

Variables	All patients		Patient with ICS use	Without ICS use	p-value
	n	(%)	n (%)	n (%)	
Age group					
0∼≤3.12 y	2684	49.89	568 (46.10)	2116 (51.01)	0.0025^*^
>3.12 y	2696	50.11	664 (53.90)	2032 (48.99)	
Gender					
Female	2071	38.49	434 (20.96)	1637 (79.04)	0.0073^*^
Male	3309	61.51	798 (24.12)	2511 (75.88)	
Income (NTD)					
<15840	5298	98.48	1217 (22.97)	4081 (77.03)	0.2171
≥15840∼<21900	62	1.15	9 (14.52)	53 (85.48)	
≥21900	20	0.37	6 (30.00)	14 (70.00)	
Medical disease					
Prematurity (ICD 765)	29	0.54	9 (31.03)	20 (68.97)	0.2958
Myopia (ICD 367.1)	3051	56.71	725 (23.76)	2326 (76.24)	0.0846
Cataract (ICD 366)	24	0.45	5 (20.83)	19 (79.17)	0.8092
Diabetes (ICD 250)	47	0.87	11 (23.40)	36 (76.60)	0.9341

a) ICD, The International Statistical Classification of Diseases and Related Health Problems, 9th Revision; ICS, inhaled corticosteroids; NTD, New Taiwan Dollars.

b) ^*^p<0.05.

### A lower incidence of glaucoma seen in the ICS cohort than in the non-ICS cohort

**Figure 1:**
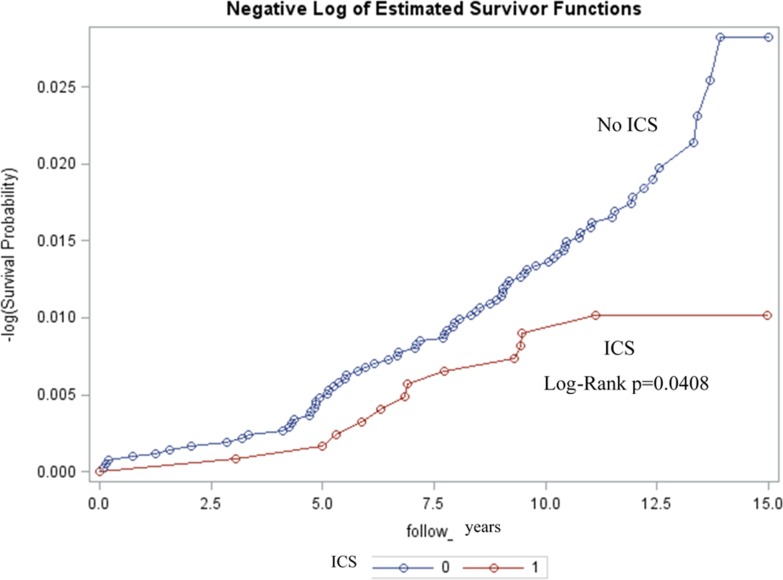
Kaplan-Meier estimates showing lower rates of glaucoma among patients with ICS (inhaled corticosteroid) (p = 0. 0408)

By the end of the follow-up period, 12 patients in the ICS group and 76 in the control group had developed glaucoma. A reduced risk of glaucoma was observed in children treated with ICS compared to the group not treated with ICS during the follow-up period (HR = 0.54, 95 % CI = 0.29∼0.98) (Table 2). Once we adjusted for certain comorbidities, age, gender, and socioeconomic status, the risk of glaucoma in the asthma group treated with ICS remained low (HR=0.52, 95% CI=0.28-0.96). The Kaplan-Meier survival analysis revealed a significantly lower cumulative probability of glaucoma among ICS users when compared with non-users during the follow-up period (Log-Rank p=0.0408) (Figure 1).

**Table 2:** Correlation between comorbidities and the risk of developing glaucoma among asthma children

	No. of patients	No. of person-years	No. of patients with glaucoma	Incident rate (per 100,000 person-years)	Crude HR (95%CI)	Multivariate-adjusted HR^*^ (95% CI)
Age group						
∼≤3.12 y	2684	32315.49	29	89.74	1.00	1.00
>3.12 y	2696	32105.40	59	183.77	2.07 (1.33∼3.23)^*^	2.09 (1.34∼3.27)^*^
Gender						
Female	2071	24689.38	41	166.06	1.00	1.00
Male	3309	39731.51	47	118.29	0.71 (0.47∼1.08)	0.73 (0.48∼1.11)
Income (NTD)						
<15840	5298	63371.18	87	137.29	1.00	1.00
≥15840∼<21900	62	790.44	1	126.51	0.88 (0.12∼6.32)	0.58 (0.08∼4.19)
≥21900	20	259.27	0	0.00	--	--
ICS use						
No ICS use	4148	49696.16	76	152.93	1.00	1.00
Any ICS use	1232	14724.73	12	81.50	0.54 (0.29∼0.98)^*^	0.52 (0.28∼0.96)^*^
Medical disease						
Prematurity						
Without Prematurity	5351	64082.85	88	137.32	1.00	1.00
With Prematurity	29	338.03	0	0.00	--	--
Myopia						
Without Myopia	2329	27819.58	33	118.62	1.00	1.00
With Myopia	3051	36601.3	55	150.27	1.27 (0.82∼1.95)	1.20 (0.78∼1.85)
Cataract						
Without Cataract	5356	64146	85	132.51	1.00	1.00
With Cataract	24	274.88	3	1091.39	8.18 (2.59∼25.87)^*^	8.22 (2.59∼26.12)^*^
Diabetes						
Without Diabetes	5333	63835.12	87	136.29	1.00	1.00
With Diabetes	47	585.76	1	170.72	1.23 (0.17∼8.82)	1.28 (0.18∼9.17)

a) CI, confidence interval; HR, hazard ratio; ICS, inhaled corticosteroids; NTD, New Taiwan Dollars.

b) ^*^p<0.05.

### Factors that may affect the development of glaucoma in asthma patients

Patients with cataract were at a greater risk of developing glaucoma (adjusted HR, 8.22; 95% CI, 2.59-26.12). The incidence of glaucoma in patients in the non-cataract and cataract groups were 132.51 and 1091.39 per 100,000 person-years, respectively (Table 2). On the other hand, multivariate analyses indicated that premature birth was not correlated with an increased glaucoma risk. The glaucoma incidence for myopia and non-myopia patients in the sample were 55 and 33, respectively, for an incidence rate of 150.27 and 118.62 per 100,000 person-years. Only one child with asthma and glaucoma was found among the 47 diabetic patients. We did not observe any correlation between myopia or diabetes and developing glaucoma during the follow-up period (adjusted HR 1.20; CI=0.78-1.85; adjusted HR 1.28; CI=0.18-9.17, respectively).

## DISCUSSION

In this population-based cohort study, we found that preschool children with asthma prescribed ICS had a significantly lower likelihood of developing glaucoma. Nevertheless, further research must be carried out with regard to the mechanisms for the reduced glaucoma risk associated with ICS use.

Glaucoma is a complex neurodegenerative disease that causes blindness. Many studies have shown that glaucomatous neuropathy may involve immunological factors [10, 11]. For example, one previous study found that serum interleukin (IL)-4 and IL-6 cytokines produced by T helper cell 2 were elevated in glaucoma patients; these cytokines also activate B cells to produce IgE [12]. In a retrospective cross-sectional study of 1,652 participants in the National Health and Nutrition Examination Survey (NHANES), the authors concluded that sensitivity to cat and cockroach antigens were associated with an increased likelihood of developing glaucoma [13]. Rubbing one's eyes is another common sequelae of allergic inflammation and has been indicated as a cause of optic neuropathy that can imitate the mechanism for glaucoma [14]. As with allergic rhinitis, asthma has a high comorbidity with allergic conjunctivitis (AC) [15]. Allergic conjunctivitis occurs frequently in preschool children and even more so in preschool children with asthma and allergic rhinitis [16]. Atopic keratoconjunctivitis and vernal keratoconjunctivitis (VKC) involve both IgE and non-IgE mechanisms. A severe bilateral chronic allergic inflammatory disease of the ocular surface, VKC has been reported to be associated with asthma. Continuous uncontrolled inflammation can result in permanently reduced or a complete loss of vision in children suffering from VKC. Topical corticosteroids are among the most potent pharmacologic agents used to treat the more severe variants of ocular allergies, as well as acute and chronic forms of AC [17, 18].

Corticosteroids can reach the eye via several administration routes. Intranasal corticosteroid sprays have demonstrated an additional advantage in reducing the symptoms of ocular allergies [19]. Currently, data related to the ocular effects of inhaled corticosteroids is limited [20]. ICS reduce airway inflammation and hyper-responsiveness, as well as their symptoms and severity, and prevent or reduce the occurrence of acute asthma exacerbations [2, 20]. Early childhood wheezing and asthma are heterogeneous disorders [1]. Respiratory viral infections, such as rhinovirus or respiratory syncytial virus, contribute to the development and exacerbation of asthma and have been positively correlated with the hospitalization of children with asthma [21]. A meta-analysis of preschoolers with recurrent wheezing and asthma demonstrated that daily ICS therapy could reduce exacerbations by nearly 40% [22]. Therefore, potential mechanisms that associate a reduced risk of glaucoma with the use of ICS may involve the inhibition of chronic inflammation. ICS significantly decreases the need for oral corticosteroids in patients with severe asthma, thus indicating a decrease in systemic adverse events like glaucoma [19]. The various anti-inflammatory pharmacological mechanisms ascribed to ICS are the most likely causes for ICS's effectiveness on asthma. ICS has extensive anti-inflammatory properties to reduce airway, lung, and systemic inflammation. Most systematic absorption of ICS occurs through the lungs. Capable of reducing peripheral blood T cell activation and 'Th2-type’ cytokine mRNA expression, ICS therapy used to treat childhood asthma possesses immunosuppressive and anti-proliferative properties [23]. In one study involving childhood asthma, ICS treatment was found to decrease the blood eosinophil cationic protein concentration [24]. Another study of adults with chronic persistent asthma reported that ICS usage significantly reduced sputum and blood eosinophils [25]. Asthmatic patients in that study had various changes in biomarker levels after ICS therapy: 82% had a decreased fraction of exhaled nitric oxide values, 60% had decreased sputum eosinophil counts, and 58% had decreased urinary bromotyrosine levels (a biochemical fingerprint of eosinophil activation) [26]. Furthermore, nationwide population-based cohort studies have demonstrated that ICS can help protect against lung cancers and osteoporosis in patients with chronic obstructive pulmonary disease [20, 21]. The population-based prospective studies showed that among the prescription of antiasthma agents for children, ICS were used in 3.1-11.0% of patients with asthma in Taiwan, with the rate varying by patient age and severity [27–29]. In this study, we also found that ICS was not frequently prescribed. The percentage of preschool-age children prescribed inhaled formulations was 22.9%, while the other 77.1% were not prescribed ICS.

Worldwide, cataract is among the leading causes of visual disability in children [30]. The available literature has already found that children with congenital diseases (e.g., Marfan syndrome, Lowe syndrome) are at an increased risk for glaucoma and early cataract formation [31]. Therefore, physicians should pay attention to genetic background when dealing with these conditions. Furthermore, while secondary glaucoma is the most troubling complication of pediatric cataract surgery, previous studies have not yet been able to determine a definite association between glaucoma and cataract within the cohorts investigated. Interestingly, in this nationwide population-based study, we found that cataract patients were at an 8.22-fold increased risk of developing glaucoma. As a result, the intraocular pressure in this particularly high-risk group requires additional research.

Various studies have demonstrated a correlation between diabetes mellitus and glaucoma [32, 33]. Furthermore, many cases of myopia have been associated with an elevated likelihood of developing glaucoma [34]. However, no significant difference has been found between the myopia or diabetes groups and the control groups in preschool-age children with asthma. Statistical significance may be lacking due to a few different factors. First, the sample sizes of ICS users and glaucoma cases were small, thus resulting in a lack of statistical power. Second, school-age children and children without asthma were excluded from this study, creating a selection bias that may have affected the results. The diagnoses of glaucoma, asthma, and other comorbid diseases were mainly based on ICD-9-CM codes, another limitation. Furthermore, information on the severity of visual dysfunction and objective measures of visual impairment was lacking. We were also unable to overcome the immortal time bias in this study design, which is another potential weakness, as were the challenges in removing bias from immortal time. To prevent the methodological issue of the immortal time bias, which consists of the time between inclusion and the moment of first starting the ICS, more research is necessary in order to develop methods to remove immortal time bias [35]. The median cumulative dose of ICS was 23 mg, and no significant correlation was found between the cumulative dose (> 23 mg compared with ≤ 23 mg) of ICS and glaucoma. The incidence rate of glaucoma and cumulative dose of ICS are likely too low to examine the dose-effect relationships of glaucoma based on the ICS dose-stratified subgroups.

This study is likely the first to show that ICS use among preschool-age children with asthma is correlated with a significantly reduced glaucoma incidence. Clinicians should be particularly careful when dealing with asthmatic children with cataract as they have a significantly increased risk of subsequently developing glaucoma. Additional consultation with an ophthalmologist is necessary for this high-risk group.

## MATERIALS AND METHODS

### Ethics statement

The Institutional Review Board of Kaohsiung Chang Gung Memorial Hospital in Taiwan approved this study (IRB No.102-0364B). The NHIRD provided the researchers with anonymous identification numbers prior to releasing the database, so the review board could exempt the requirement for written informed consent.

### Data sources

Our data were obtained from a NHIRD subset known as the Longitudinal Health Insurance Database (LHID), which chose one million insured individuals at random from 1996 to 2000. This computerized database was derived from the Taiwan National Health Insurance Program, which was created in 1995. Said program offers universal health coverage and equal medical access to all Taiwanese citizens. Both hospitals and clinics in Taiwan are densely distributed and highly accessible and offer services at a very low cost, which motivates people in Taiwan to take advantage of such services. In 2011, the coverage rate of Taiwan's National Health Insurance was 99.6%, so indicating that nearly the entire population of Taiwan (23 million) was enrolled in the program. The database consists of patient demographic information, gender, birth date, the registry for beneficiaries, diagnosis records (based on the International Classification of Diseases, 9th Revision, Clinical Modification [ICD-9-CM]), drug codes, diagnosis dates, admission dates, and procedures.

### Determining the asthma cohort

**Figure 2:**
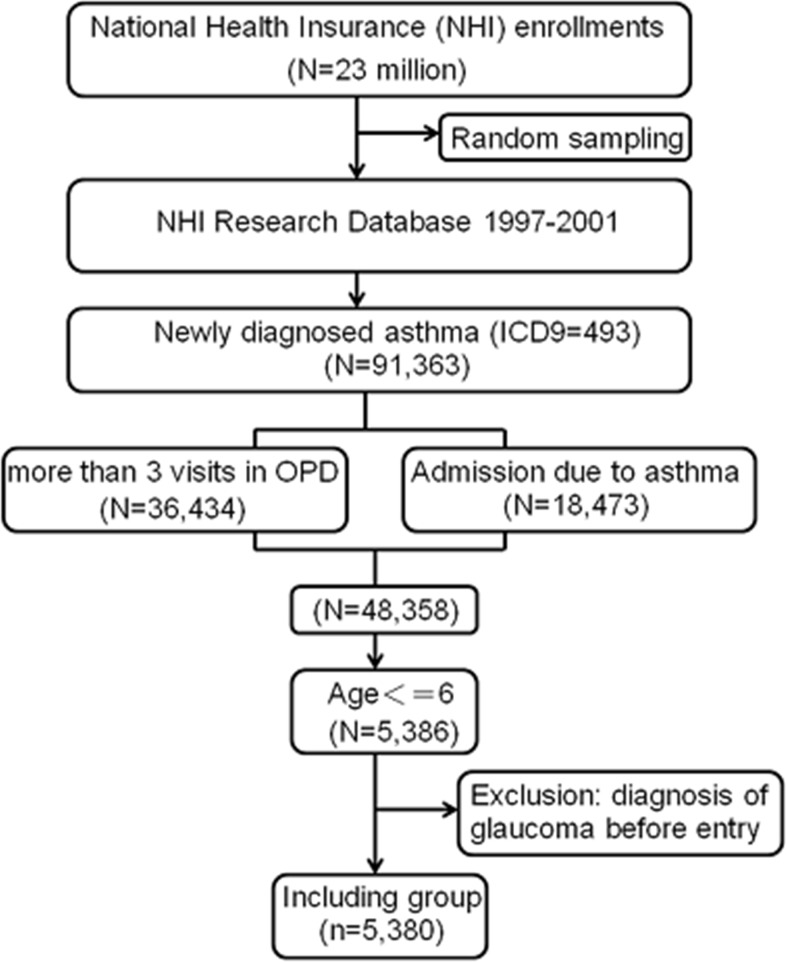
Flowchart of the enrollment process. OPD, outpatient department

Figure 2 shows the flowchart of the process we used to select the study population. A retrospective population-based cohort study was conducted from January 1, 1997 through December 31, 2001. Based on the diagnostic codes for asthma in the NHIRD (ICD9: 493.X), we identified 5,380 children six years old or younger. We restricted the population to cases of newly diagnosed asthma in the LHID (n= 91,363) and further limited the analyses to patients with at least one hospital admission for asthma or at least three outpatient clinic visits for asthma, which we considered a validated diagnostic indicator of asthma (n= 48,358). After excluding subjects over the age of six years old, this study included 5,386 patients with new-onset asthma. We also excluded patients diagnosed with glaucoma before entry (n=6), so our final asthma study cohort consisted of 5,380 children. According to their treatment therapies, we categorized patients with asthma who used ICS into the ICS group patients who had never used ICS into the unexposed group.

We chose certain demographic factors, like gender, age of asthma diagnosis, and comorbidities, to determine the potential risk factors related to glaucoma in children with asthma. We collected such data as prematurity (ICD-9-CM 765), myopia (ICD-9-CM 367.1), cataract (ICD-9-CM 366), and diabetes (ICD-9-CM 250) to adjust for the risk of glaucoma, focusing on the development of glaucoma as the relevant outcome. Patients diagnosed with glaucoma were identified from the database using the code 365.X in the ICD-9-CM format. The study's endpoint was the development of glaucoma. All children in our study cohort who did not develop glaucoma were followed through the end of 2011. Therefore, a sensitivity analysis was performed in which we excluded these patients who started ICS after a glaucoma diagnosis from our analysis.

### Statistical analysis

To study the risk of asthma children developing glaucoma, descriptive statistical data of both ICS users and non-users are presented as a number and percentage for age group and comorbidities. We divided the case number from glaucoma by the number of person-years of follow-up to determine the incident rates. To define the correlation between ICS usage and potential risk factors, we estimated the hazard ratio (HR) and 95% confidence interval (CI) using Cox proportional regression models. Furthermore, adjusted HR was corrected for potential bias sources. Results were considered statistically significant if *α* *=* 0.05. Data management and analysis were carried out using SAS 9.3 software (SAS Institute Inc, Cary, NC, USA).

## Abbreviations

CI: confidence interval
HR: hazard ratio
ICS: inhaled corticosteroids
LHID: Longitudinal Health Insurance Database
NHI: National Health Insurance
NHIRD: National Health Insurance Research Database
